# A Treatise on Head-Aches, Their Various Causes, Prevention, and Cure

**Published:** 1835-10-01

**Authors:** 


### A Treatise on Headaches, their various Causes, Prevention, and Cure. By *George Hume Weatherhead*, M.D. &c. &c.

After the laborious and erudite volume of the late Dr. Vaughan, of Rochester, on the subject of headaches, we did not expect to find much novel matter in this small duodecimo of Dr. Weatherhead's, though we were quite aware of the talent for observation possessed by the author. We were agreeably disappointed—and the cause may, very probably, be ascribed to the circumstance of the author being himself a sufferer from the complaint. None can describe a disease so well as he who has felt it, *cæteris paribus.* Hence we have had some excellent monographs from the pens of physicians, who delineated from their own sufferings.

Dr. W. has prefixed to the more practical part of his subject, a very well-written chapter on the physiology of the brain. On this we need not dwell. But we are tempted to make an extract from this chapter, respecting the difference between reason and instinct—and the cause of that difference.

" All former physiologists and psychologists have failed in their attempts to bring the instinctive faculties under those proper to the brain. The wonderful works of bees and spiders would suppose a degree of intelligence greater than even the higher and more docile classes of the vertebrated animals ever attain to, superior even to what man, as an isolated being, could evince, were he to try to execute any thing so geometrically perfect.

Thus naturally led to the reflection, let us now observe the contradiction which such a train of ideas involves. It is an accorded fact, that the insect tribe are infinitely below the humblest of the vertebrated animals in relation to the size of the brain; and yet, if their instincts are acts of this organ, how comes it that many of them should be so superior in this respect to most of the vertebrated animals? Comparative anatomy solves the question—they are acts which the brain does not execute. From the foregoing observations, therefore, we are enabled to infer, that man ranks in the lowest grade as an instinctive being, because he is the most inferior of all relatively to the proportional volume of the fifth pair of nerves compared with the brain. The bee, again, may be placed at the head of the invertebrated animals for its instinctive faculties, because its cranial ganglions are the most developed.

If it were necessary to adduce proof of the exceeding value of physiological knowledge, in elucidating what hitherto has afforded to the materialist and sceptic a tenable position for argumentation, founded on the mental manifestations of the brute creation, and what has no less evaded the researches and speculations of the profoundest metaphysicians satisfactorily to explain,—no more cogent or conclusive example could be given, than the manner in which the investigations of comparative anatomy have demonstrated the difference of the organs of instinct and of mind." 18.

Headache is, of course, only a symptom of some disease, and, consequently, is always symptomatic—never idiopathic. The following is the order in which our author proposes to treat of this class of complaints.

"I. Dyspeptic, or Sick Headache.
II. Nervous Headache.
III. Headache from Fulness of Blood in the Head; originating from
Venous Plethora,
Arterial Plethora.
IV. Rheumatic Headache.
V. Arthritic Headache.
VI. Headache from Organic Lesion of the Brain." 20.

The first variety is not the most common form, though it is generally considered so both by patient and practitioner. The doctrines of Abernethy and Philip have led people to refer almost every complaint of the body to disorder of the digestive organs. There can be no doubt that these organs are very frequently affected, either primarily or secondarily, in a great number of maladies; but, as a certain personage is not so black as he is painted, so the stomach and liver are often blamed, when they are more sinned against than sinning.

The dyspeptic or sick headache, however, is described with great truth and clearness by Dr. Weatherhead—more faithfully, indeed, than by any author that we are acquainted with.

"It is usually ushered in by a sensation of chilliness passing over the frame, and a feeling of fatigue and lassitude pervading the whole muscular system. While the feet are cold, the countenance is often flushed and tumid, and the forehead burning hot. A pain, varying in character, is felt most usually over the forehead, or in one or other of the temples. At times, this pain is dull and heavy, attended with much heat, and a sense of weight and fulness, as if the bloodvessels were over-gorged, which, indeed, is the fact; the temporal arteries beat with violence, imparting a throbbing character to the pain, aggravating it by each pulsation, while all the veins about the head (most observable on the forehead) are swollen and distended. There is a total prostration of appetite, and sickness is felt at stomach; the pulse is quickened, often full; the tongue coated with a brown fur; an acrid sensation is felt in the back part of the throat, particularly after eructations; the mouth is clammy; the saliva viscid; the breath offensive; the skin is dry and parched; the urine is usually limpid and abundant; the palms of the hands are burning hot; and in many cases the feet are icy cold.

During the continuance of the pain, the patient is incapable of applying his attention to any thing; his thoughts are distracted and confused, and his recollection greatly impaired. Indeed, the common effect of habitual headachs is to injure the energy of the intellect, and especially the memory.

The pain, if seated in the temple, will not unfrequently shift into the eye-ball of the same side, or it will fix itself over the inner corner of the eye-brow, affecting, in fact, the supra-orbitar branch of the fifth pair of nerves, as it emerges from the orbit to spread its ramifications on the brow. This particular kind of headach is, by the general consent of medical writers, attributed to gastro-intestinal irritation, proceeding from acrid acidity in the stomach and duodenum; and no headach is more excruciating: the sufferer seeks quietude and silence, being distracted by company;—the least noise aggravates the pain, and light is painful and oppressive to the eyes, contracting the pupil.*

With the above symptoms there is usually conjoined much sickness at stomach, which is sure to be increased by erect posture, or by moving about. At length retching comes on, and speedily terminates in vomiting, attended with violent strainings. The matter usually first ejected consists of the contents of the stomach in an undigested, or corruptly digested state, mixed, for the most part, with much acrid acidity; sometimes, indeed, the matter brought up consists of nothing else; but if the retching and straining continue, bile is at length ejected—a result not always to be attributed to any undue quantity in the secretion itself, but rather to the pressure which is made by the muscles, in the action of vomiting, on the gall-bladder, emptying it of its contents, and forcing it into the stomach. We thus see that the familiar appellation of 'bilious' as applied to headachs is often used incorrectly, the presence of bile being entirely incidental; in many instances, the *effect* of severe straining, and not the *cause* of the headach." 23.

The stomach being freed from its contents, and all the emunctories having been opened, the pain usually abates—sleep often succeeds—and the patient generally awakes free from headache.

Dr. W. thinks that these headaches are sometimes occasioned by atmospheric influence, since they occur where there has been no "irregularity or imprudence over-night." But we can assure Dr. W. that irregularities in diet are by no means necessary to induce indigestion. Passions of the mind will disorder the stomach far more effectually than wrong food—hence the great number of dyspeptics who observe the most exact regimen. We are rather surprised that Dr. W. has excluded, or at least omitted, moral causes of sick headache from his etiological catalogue. The common or material causes he has correctly enumerated, and we need not repeat them. The treatment he defers, till the second variety—the nervous headache, is under consideration. The temperament which disposes to this species, both in males and females, is indicated by variable spirits—fickleness of temper—and excessive susceptibility to all impressions, moral and physical.

"The mind and body reciprocally act upon each other in all constitutions, but they are more especially sympathetic in the nervous; and as physical agents, acting through the corporeal sensibility affect the mind, so mental emotions and impressions no less powerfully operate upon the nervous system." 29.

Atmospheric influence, Dr. W. thinks, is one great physical cause of this kind of headache.

"It not unfrequently happens that a nervous headache, which has continued severe through the day, goes off in the evening. This is sometimes attributable to a change in the state of the atmosphere itself; but is, perhaps, frequently better accounted for by ascribing it to that *periodical augmentation which takes place in the energy of the brain and nervous system generally, recurring every even-*

* "Meckel explains this last symptom by the conjunction of the first branch of the fifth and the third nerve with the ophthalmic ganglion, from which the ciliarly nerves proceed."

*ing*. It is this diurnal revolution which relieves the symptoms, for a time, of all adynamic diseases, and which so regularly aggravates those of an opposite character; imparting an excitement which dissipates, while it lasts, the gloomy visions of the hypochondriacal, and which produces the vesper exacerbations and delirium in fevers." 35.

We confess that the foregoing explanation appears to us very questionable —and we have no doubt that it will give foundation to a long "critical disquisition," in the new aspirant for critical fame about to appear above the horizon. For our own parts, we shall merely enter our dissent against the doctrine of *augmentation of energy in the brain towards evening*. We suspect that it is just the reverse—and that if Dr. W. tries the experiment, he will find his own brain more energetic in the morning, after a good night's rest, than in the evening, when the whole corporeal and mental powers stand in need of recruit. We are aware, indeed, that many people invert the order of nature, and, by sitting up till 2 or 3 o'clock in the morning, under the influence of artificial excitement, they induce languor and low spirits all the forenoon of the next day—not rallying till after dinner and tea in the evening. But this is a violence done to nature, and, indeed, it is the usual course which ultimately leads to nervous and hypochondriacal complaints.

The various forms of hysterical headache come under this class—the most remarkable of which is the clavus hystericus.

"A tendency to headach not uncommonly originates in some latent source of irritation, which, by the sympathy of the nerves, is transmitted to the head. One of the most usual seats of this irritation is the alimentary canal; and there are three principal sources of it: *firstly*, acrid secretions working on the sensibility of the inner membrane of the intestines; *secondly*, accumulation of indurated fæces from habitual constipation; and *lastly*, worms. The last is more especially to be suspected as the cause of headach when it happens and frequently recurs in children. Worms act on the head in a manner analogous to acrid sordes in the first passages, that is, by sympathetic irritation; and when the worm is a tænia, the disease is often accompanied with convulsions. Hoffman (tom. iii. p. 42) gives a case of this kind where the patient suffered for four years from this cause, without its true nature having been suspected; and I have in my own practice met with more than one case of a similar character. But, taken generally, habitual constipation is by far the most common cause. Hence headach from a sluggish state of the bowels is the prevailing disorder of the sedentary, and is very justly noted as a 'desk disease.' A morbid degree of sensibility of the whole frame, rendering it susceptible of slight impressions, sometimes owes its origin to a source often little suspected for a length of time—I mean the urethra. Stricture of some part of this canal I have frequently traced as the latent cause of great constitutional disturbance, of much morbid susceptibility, and as an indirect cause of habitual headach." 37.

Our author notices a nervous headache, resulting from debility or exhaustion. It is accompanied by vertigo, and a sense of falling down, together with great sensibility to the slightest annoyances—the appearance of a veil or network, full of dark moving spots, before the eyes—unsteadiness of thought, fickleness of temper, and despondency. This is a very obstinate form of headache, and most difficult of cure.

The treatment of sick headache and nervous headache Dr. W. divides into two clauses—the palliative and systematic. When arising from crudities in the stomach, they ought to be dislodged, and acidities corrected. Dr. W.

has often found quick relief from a wine-glassful of peppermint water, with a little magnesia and rhubarb. But emetics or purgatives give the most effectual relief. Warm water of chamomile tea alone will often dislodge the enemy.

For the radical treatment of the biliary headache, Dr. W. recommends a course of blue-pill and colocynth, with saline waters, as those of Cheltenham and Beulah. The quantity of saline ingredients in the waters of Beulah is greater than in those of Cheltenham. As these headaches generally depend on deranged function in one or more of the digestive organs, our attention must be almost entirely directed to that source. Great restriction in diet—confinement, for example, to meat and bread, with weak brandy and water—regular, but mild aperients—with alkaline bitters, will do more to remove these headaches, and improve the general health, than all the remedies in Ryan's conspectus.

Dr. W. makes some judicious remarks on headaches from plethora in the brain, and especially in the veins of the head; but we do not deem it necessary to quote them, with the exception of the following passage.

"Headach from fulness of blood in the veins of the brain is apt to occur in men of literary pursuits, and in those whose business confines them to the desk, or to a constant stooping position of the head: these, when conjoined with sedentary habits, and intense application of the mind to some harassing occupation, all concur to derange the equilibrium of the circulation. The first effect is to throw the blood in undue quantity to the head: in time this determination becomes habitual; the brain is exhausted by the intensity and long continuance of the excitement, and the consequence of this exhaustion is congestion in the veins: the healthy tone of the vessels is destroyed; and when this state of engorgement has attained a certain height, some accidental aggravation of it produces rupture of one or more of the vessels, and the patient has an apoplectic or paralytic seizure." 57.

On rheumatic headaches Dr. W. does not much dilate. They are much more numerous than most practitioners apprehend, and are often mistreated. We have found no means so beneficial as the following:

℞. Pulv. Ipecac. c........... gr. viij.
Sub. Hyd................. gr. ij.
Ft. Pulv.—Hora somni sumend.

℞. Infusi Rhei ............. ℥iij.

Tart. Sodæ ............. ʒiij.

Pulv. Rhei............... ʒss.

Tinct Sennæ.............. ℥ss.

Vini Colchici............ ʒjss.

Misce ft. Mistura, capiat tertiam partem prima mane, et repr. dosis alternas horis donec alvus plena respondeat.

If these doses be repeated every second night and morning for two or three times, they will generally remove the disease.

From the 5th section on "ARTHRITIC HEADACHE," we make the following extract.

"Gout affecting the head is one of the most dangerous forms of the disease, and shews itself either as arthritic headach, or as arthritic apoplexy; indeed, the one is the usual precursor of the other. The first is a disease that often escapes detection, especially if the patient has never had a paroxysm of the gout in its regular form: the symptoms are imputed to a common determination of blood to the brain, and treated accordingly; but this is a mistake of a serious nature, since, in the treatment of gout affecting the organ, all depletory measures are hurtful. Hence it is a disease frequently requiring both considerable discrimination and close observation to detect, as it is only by apparently trivial distinctions that we are enabled to determine its true nature. This form of the disease affects, for the most part, only those in whom gout does not make a demonstration in the open and regular way. It is most frequently to be met with among the higher classes of the community, where this heir-loom of luxury can frequently claim lineal descent on the part of both parents; and it is likewise found in this disguised form prevailing more among the females of a gouty family than the males, and shews itself particularly after a certain period of life. The most pathognomonic of the symptoms characterising arthritic headach are, a constant sense of fulness in the head, liable to change, from casual circumstances, into severe pain; much giddiness, and a feeling of movement within the head, which keeps continually attracting attention to the sensation, rendering thought confused, and memory forgetful; and at the same time there is often a feeling experienced by the patient as if he were to become insensible: when he stoops, he is apt to be seized with temporary blindness; his hearing is inordinately acute, he is distracted by the least noise, and he is frequently troubled with a constant buzzing in his ears; flushing and heat pass at times over the head and face, the capillitium is often tender to the touch, and the head feels constantly hot and uncomfortable; digestion is often much disordered, the bowels slow, and the evacuations discoloured; the urine is usually scanty, high-coloured, depositing a reddish sediment; and yet, in the midst of so much disordered feeling and function, the pulse, for the most part, remains undisturbed.

The above are the symptoms most characteristic of this specific form of headach, and which, if not removed, are sure to terminate in arthritic apoplexy." 87.

One more extract, and we take leave of this little volume.

"As the most efficacious palliative in any extraordinary urgency of symptoms, we are to have recourse to colchicum, taking care to direct its operation gently on the bowels, by the addition of some sulphate of magnesia; the circulation ought likewise to be solicited to the feet by means of a pediluvium, made stimulant by a table-spoonful or two of mustard flour; and a gentle diaphoresis brought out on the surface by James's powder taken in white wine whey, or some other mild sudorific. But there is an effect produced by colchicum, besides its anodyne operation, that involves certain pathological questions respecting the real nature of gout, which have not been hitherto satisfactorily elucidated—I allude to the effect which colchicum has of augmenting *the quantity of uric acid in the urine.* There are various circumstances which prove that this acid, or its base, urea, superabounds in the blood of gouty people; the result, as I conceive, of a morbid condition of the function of sanguification, or rather, perhaps, of the immediate process of chylification. Now, Chelius of Heidelberg has ascertained that the quantity of uric acid excreted by the kidneys is nearly doubled in twelve days under the use of colchicum.

Another curious circumstance which presents itself in the consideration of this subject, is that *tofi*, or gouty chalk-stones, as they are more commonly called, do not consist, as was once imagined, of phosphate of lime, but of *urate of lime*. I may further notice, that in the decline of every fit of the gout, *uric acid* is always observed to abound in the urine of the patient; and in conclusion I may remark, that the use of alkalis and other antacids has an evident influence in diminishing the formation of urea; while they are found, at the same time, to be among the best preventives of gout. Now, the probable inference from all the foregoing facts, is, that gout is in reality occasioned by the superabundance of urea in the blood, generated in the intestines by an unhealthy process of digestion, induced by habits of luxury and indulgence; or it proceeds from the same cause, in consequence of the same morbid habit of the digestive organs of a hereditary nature. May not the phenomena of a fit of the gout, therefore, be considered as arising from this excess of urea; and the deposition of it in the form of chalk-stones, and its excretion by the kidneys, be regarded as modes of the salutary crisis?

The above considerations, if well founded, teach us, that though it may be proper to assist Nature, in her effort to accomplish her purpose during the fit, still, in the systematic treatment of gout, our paramount object ought to be to prevent its accession, by counteracting the cause—excess of urea." 89.

The volume concludes with some brief, but judicious hints, of a hygienic nature. In his remarks on temperance and travelling, we coincide entirely —and it is evident that he speaks from personal experience. We hope Dr. Weatherhead will pursue his investigations, and soon come before the world with the results of his lucubrations.

